# West Nile Virus Detection in Urine

**DOI:** 10.3201/eid1108.050238

**Published:** 2005-08

**Authors:** Jessica H. Tonry, Craig B. Brown, Cecil B. Cropp, Juliene K.G. Co, Shannon N. Bennett, Vivek R. Nerurkar, Timothy Kuberski, Duane J. Gubler

**Affiliations:** * Asia Pacific Institute of Tropical Medicine and Infectious Diseases John A. Burns School of Medicine, Honolulu, Hawaii, USA;; † Arizona College of Osteopathic Medicine, Glendale, Arizona, USA;; ‡ John C. Lincoln Hospital Deer Valley, Phoenix, Arizona, USA

**Keywords:** West Nile virus, urine, encephalitis patient

## Abstract

We report West Nile virus (WNV) RNA in urine collected from a patient with encephalitis 8 days after symptom onset. Viral RNA was detected by reverse transcriptase–polymerase chain reaction (RT-PCR). Sequence and phylogenetic analysis confirmed the PCR product to have ≥99% similarity to the WNV strain NY 2000-crow3356.

West Nile virus (WNV) is a mosquitoborne flavivirus in the Japanese encephalitis serocomplex of the family Flaviviridae (1). Human disease is typically characterized by a mild, self-resolving, denguelike illness with the onset of fever and myalgia (2,3). In a small percentage of patients, primarily the elderly and immunocompromised, disease progresses to a more severe form with central nervous system (CNS) involvement, including encephalitis and meningitis (4,5). The death rate among patients with neuroinvasive disease in recent epidemics has averaged 10%. Among survivors, long-term neurologic sequelae may occur (6). Because WNV is a neurotropic virus, its pathology in the CNS has been the focus of many studies; therefore, little is known about WNV pathogenesis in organs other than the CNS. Studies of WNV in birds, dogs, and rodents have shown that the kidney is a site of replication (7–9); moreover, infectious WNV has been recovered from urine samples from experimentally infected hamsters as early as day 1 to day 52 postinfection (9). The purpose of this study was to determine whether WNV is similarly shed in the urine of humans with WNV infections. To our knowledge, this report is the first of WNV RNA detected in the urine of an infected patient with encephalitis.

## The Study

A 65-year-old computer software engineer was admitted to a hospital in Phoenix, Arizona, on July 7, 2004, with fever, headache, and altered mental status evolving in the 7 days before admission. His cerebrospinal fluid (CSF) findings were consistent with viral encephalitis: leukocyte count 141 cells/mm^3^, 27% polymorphonuclear cells, 73% lymphocytes; glucose 106 mg/L; protein 102 mg/L; and abnormal electroencephalogram results. The patient was treated empirically for 9 days with acyclovir, ribavirin, and interferon α-2B beginning on July 7, 2004. His fever resolved, followed by gradual improvement in strength and mental function. The patient recovered and was discharged.

CSF and serum samples were obtained on July 7 and 14, 2004. CSF tested positive for specific WNV immunoglobulin (Ig) M antibodies by using a capture enzyme-linked immunosorbent assay. Paired serum samples confirmed an acute WNV infection by showing a 4-fold rise in titer from acute-phase (July 7 [day 8 of illness; day of admission to hospital]) to convalescent-phase (July 14) sera on a 90% plaque reduction neutralization test (PRNT) (10). Antibody titers for acute-phase and convalescent-phase serum samples were 1:80 and ≥1:320, respectively. PRNT tests were negative on urine samples obtained on days 8 and 15 after symptom onset. CSF was unavailable for PRNT and isolation.

Attempts at virus isolation, by using Vero cells (green monkey kidney cells) and C6/36 cells (*Aedes albopictus*), from urine samples collected on days 8, 11, 12, 13, 14, and 15 after symptom onset were unsuccessful. Similarly, we were unable to isolate virus from serum samples collected on days 8 and 9 after symptom onset. Indirect immunofluorescence assays used to confirm culture results were negative.

RNA was extracted from 140 μL of freshly thawed urine by using the QIAamp Viral RNA Mini Kit (Qiagen, Valencia, CA, USA) according to manufacturer's instructions. To detect specific WNV gene sequences, we used the following primer sets: WNV 233, 640c and WNV 9483, 9794 that amplify the cap/prM gene and the NS5 gene regions, respectively (11). To generate an amplicon we used Qiagen One-Step RT-PCR Kit with the following thermocycling conditions: reverse transcription (RT) at 50°C for 20 min, 94°C for 15 min, and 55°C for 30 s; 40 cycles of polymerase chain reaction (PCR) at 95°C for 10 s, 56°C for 10 s, and 72°C for 15 s. The urine sample collected on day 8 of illness tested positive with the aforementioned primer sets. The urine specimens analyzed by RT-PCR on days 11, 12, 13, 14, and 15 tested consistently negative with both primer sets.

Amplicons generated with primer set WNV 233, 640c were purified by Qiagen Gel Purification Kit according to the manufacturer's instructions and sequenced on an automated sequencer (Model 373A, Applied Biosystems, Foster City, CA, USA). Sequencing results based on the capsid/prM region from the patient's day 8 urine confirmed the identity of WNV Arizona JW 2004 (GenBank accession no. DQ011267), which had 99.7% homology to the WNV strain NY 2000-crow3356 (GenBank accession no. AF404756.1) over the cap/prM region.

A phylogenetic tree was generated by comparing the WNV Arizona JW 2004 sequence against 34 other WNV strains by using a 356-bp sequence corresponding to nucleotide positions 260–615 in the cap/prM region (Figure). Previously published WNV isolates used in the analysis included the first WNV isolate from Uganda in 1937 (GenBank accession no. M12294), the Egypt 1951 laboratory strain (Eg101, GenBank accession no. AF260968), and several American isolates, such as the NY 2000-crow3356 isolate. After sequence alignment with ClustalW, a phylogenetic tree was constructed by using a maximum likelihood (ML) algorithm implemented in PAUP* (v. 4.0b10, Sinauer Associates, Inc., Sunderland, MA, USA). The ML tree was estimated by using the general-time reversible (GTR+I) model of nucleotide substitution, with the substitution matrix, base composition, and proportion of invariant sites (I) all estimated from the data. For tree topology support, we performed 1,000 bootstrapped neighbor-joining replicate trees under the ML substitution model described above and also generated posterior probabilities for each node by using Bayesian MCMC (Metropolis-Hastings Markov chain Monte Carlo) tree sampling (variable substitution rate by codon position, 4 chains of 2 × 10^6^ generations sampled every 100 generations, burn-in of 2,000, and convergence assessed at effective sample size [ESS] >400) implemented in MrBayes v. 3 (12).

**Figure:**
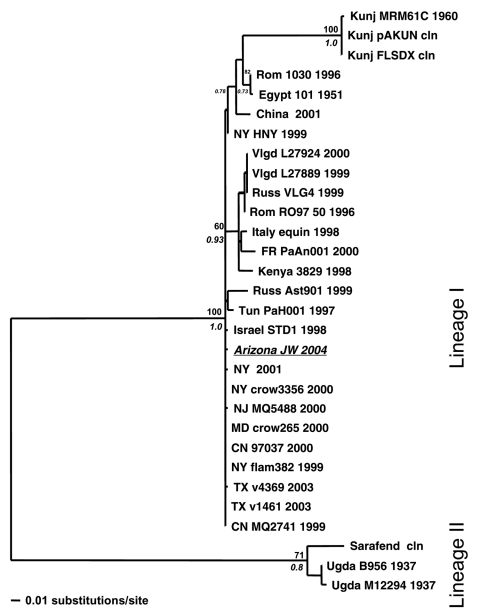
Maximum likelihood (ML) tree showing the phylogenetic relationships between West Nile virus (WNV) urine sample Arizona JW 2004 (italicized and underlined) and previously published WNV strains based on capsid/prM gene junction (356 bp). Samples are coded by location, strain, and year of isolation. Locations include France (FR); Kunjin (Kunj); Romania (Rom); Russia (Russ); Tunisia (Tun); Uganda (Ugda); Volgograd, Russia (Vlgd); and the US states of New York (NY), Texas (TX), New Jersey (NJ), Maryland (MD), and Connecticut (CN). Support indicated above and below nodes are bootstrap values (1,000 neighbor-joining replicates using the ML model of evolution) and Bayesian posterior probabilities (Bayesian MCMC [Metropolis-Hastings Markov chain Monte Carlo tree sampling] for 4 chains length 2 × 106, sample frequency 100, with a 2,000-tree burn-in), respectively.

Four main geographic groupings are observed in the phylogram. As expected, the WNV Arizona JW 2004 sequence grouped with the other isolates from the United States. Isolates from Russia, Romania, France, and Italy formed a distinct cluster, as did 3 Kunjin virus isolates and their close relatives from China and Egypt (Figure). The WNV 1937 Uganda sequence, along with other sequences (lineage II), was characterized by having the longest branch length (Figure), which implies poor homology with the other isolates.

Contamination of samples within the laboratory is highly improbable. First, the positive control used in all tests was WNV strain Eg101, isolated from Egypt in 1951. Sequence results of this control confirmed its identity and showed 96.9% homology to the cap/PrM region. Second, at the time of testing, our laboratory did not possess any North American WNV isolates, including the WNV strain NY 2000-crow3356, to which the WNV Arizona JW 2004 sequence showed 99.7% homology. Finally, sample contamination during RNA extraction and RT-PCR procedures was unlikely because we used a continuous single-sample RNA extraction, and RT-PCR was accompanied by a negative water control. Results from the continuous single-sample test confirmed previous results: the patient's urine on day 8 after symptom onset tested positive for WNV RNA by RT-PCR, and the water control was negative. No blood was visible in the day 8 urine sample. A contemporaneous serum sample collected on day 8 was negative by both RT-PCR and virus isolation.

## Conclusions

This report is the first of WNV RNA detected in urine from a patient with encephalitis. St. Louis encephalitis virus (SLEV), a related neurotropic flavivirus, has been reported in human urine. SLEV antigen was detected by indirect immunofluorescence, electron microscopy, and immune electron microscopy in 12 patients during the 1976 outbreak in the United States (13). Furthermore, experimental animal studies have shown that certain flaviviruses are shed in the urine. A study on Japanese encephalitis virus infection in a mouse model showed viral shedding in urine; however, viral shedding did not necessarily correlate with isolation of virus from the kidney (14). In a recent study, infectious WNV was isolated from hamster urine 52 days after initial infection despite the development of high antibody titers against WNV (9). Based on the above reports on flavivirus shedding in humans and experimental animals and our detection of WNV RNA in human urine, we believe WNV may be shed in human urine during the course of infection.

A rapid diagnostic test for flaviviral infection in humans is of clinical interest. Historically, flavivirus infections have been diagnosed by serologic tests or virus isolation (15). Several molecular techniques are available for diagnosis (11), but these tests are not readily accessible in many community medical facilities where disease is commonly reported. We believe the development of a rapid diagnostic test for human flaviviral infections is warranted.

The implications of our finding remain unclear. The presence of WNV RNA in the day 8 urine sample but not subsequent urine samples suggests that neutralizing antibodies in the blood may prevent virus excretion in the urine. WNV would likely be excreted in urine during the viremic phase of illness. Thus, future studies on WNV in human urine should emphasize early collection and testing. In addition, the effect of interferon and ribavirin on the recovery of WNV from the urine remains unknown. Studies currently under way will provide additional information on how often and for how long WNV can be found in urine samples from patients with clinical and subclinical WNV infections.
